# Supplementary material for: A novel mass assay to measure phosphatidylinositol-5-phosphate from cells and tissues
Source: Biosci Rep. 2019 Oct 21;39(10):BSR20192502. doi: 10.1042/BSR20192502 (PMC6822513; doi:10.1042/BSR20192502)
Supplement: Supplementary Figure S1A [file BSR-2019-2502_supp.pdf]

A

Starting d5-PI5P amount (in picomoles on column)	Co-effiecient of variation (% CV)
0.01875	56.26
0.0375	22.34
0.075	21.74
0.15	14.98
0.3	10.10

B (i)

(ii)

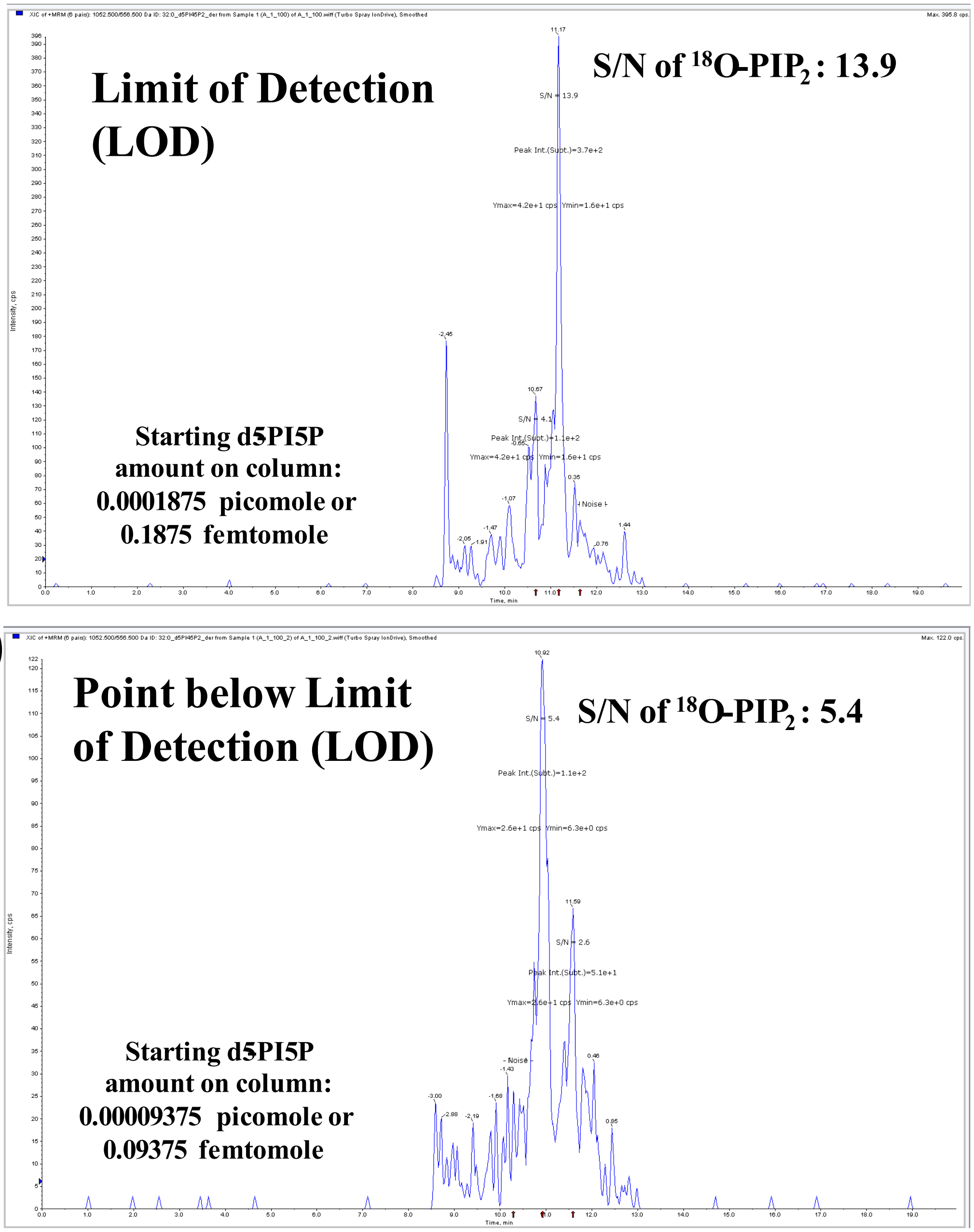

C

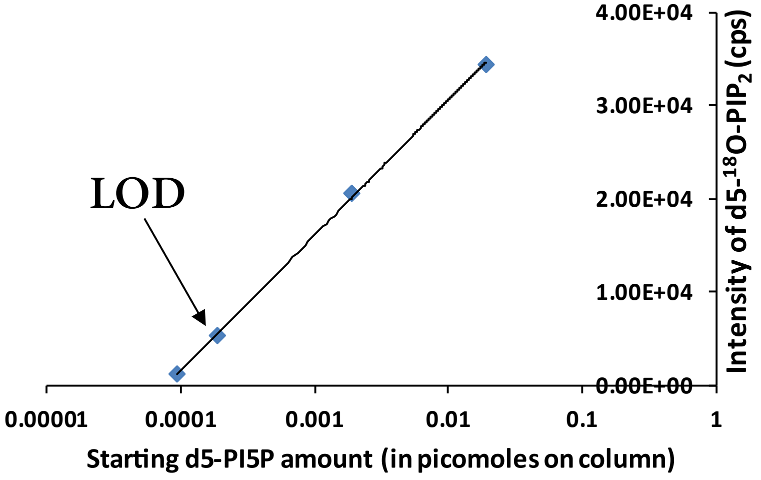

Ghosh et. al.,Supplementary Figure 1

Figure Supplementary 1: Limit of Detection analysis for ^{18}O-PIP_2 in an *in vitro* PIP4K assay

(A) CV % calculated for each point of dose response curve run in duplicates. (B) (i) Extracted ion chromatogram (XIC) of d5-^{18}O-PIP_2 (MRM transition: 1052.5/556.5) from 0.1875 femtomole starting d5-PI5P substrate. (ii) XIC of d5-^{18}O-PIP_2 (MRM transition: 1052.5/556.5) from 0.09375 femtomole starting d5-PI5P substrate. S/N was determined using Analyst® 1.6.2 software. (C) Graph representing Limit of detection (LOD) point. Y-axis depicts intensity of d5-^{18}O-PI(4,5)P_2 (in cps) and X-axis represents the amount of d5-PI5P loaded on column. Linearity equation: y = 1E + 06x + 7622.6, R^2 = 0.7787; Logarithmic equation: y = 6305.5 ln(x) + 59662, R^2 = 0.9996.
